# EFFICACY OF FOCUSED SHOCKWAVE THERAPY IN PATIENTS WITH MODERATE-TO-SEVERE CARPAL TUNNEL SYNDROME: A PRELIMINARY STUDY

**DOI:** 10.2340/jrm.v56.13411

**Published:** 2024-02-08

**Authors:** Pimpisa VONGVACHVASIN, Thitiporn PHAKDEPIBOON, Waree CHIRA-ADISAI, Punpetch SIRIRATNA

**Affiliations:** 1 Department of Rehabilitation Medicine, Maharat Nakhon Ratchasima Hospital, Nakhon Ratchasima; 2 Department of Rehabilitation Medicine, Faculty of Medicine Ramathibodi Hospital, Mahidol University, Bangkok, Thailand

**Keywords:** carpal tunnel syndrome, extracorporeal shockwave therapy, symptoms, function, electrodiagnosis, nerve conduction

## Abstract

**Objective:**

To evaluate the efficacy of focused extracorporeal shockwave therapy for symptoms and function in patients with moderate-to-severe carpal tunnel syndrome.

**Design:**

A single-blind randomized controlled trial.

**Subjects:**

Twenty-four outpatients with moderate-to-severe carpal tunnel syndrome.

**Methods:**

Patients were randomly allocated into 2 groups: a focused extracorporeal shockwave therapy group and a control group. The focused extracorporeal shockwave therapy group received conservative treatment in addition to focused extracorporeal shockwave therapy with an energy flux density ranging from 0.01 to 0.15 mJ/mm^2^, a frequency of 4–5 Hz, and 1500 pulses per session once a week for a total of 3 sessions. The control group received only conservative treatment, which comprised gliding exercises for carpal tunnel syndrome, a night wrist splint, and lifestyle modification. The Thai version of the Boston Carpal Tunnel Questionnaire (T-BCTQ), a nerve conduction study, and ultrasonography of the median nerve cross-sectional area were performed before treatment and at 3 and 6 weeks after baseline.

**Results:**

The T-BCTQ symptom and function scores had significantly decreased in both groups, favouring focused extracorporeal shockwave therapy at all time-points. In addition, distal sensory and motor latency were significantly different between the groups at 3 weeks from baseline.

**Conclusion:**

Focused extracorporeal shockwave therapy plus conservative treatment effectively provided short-term improvement in symptoms, hand function, and nerve conduction in patients with moderate-to-severe carpal tunnel syndrome compared with conservative treatment alone.

LAY ABSTRACT

Carpal tunnel syndrome is the most common compressive nerve entrapment of the upper limb. Various conservative treatment methods, including physical modalities (any therapeutic tools that convey energy to or through the patient), are used to treat this condition. Focused extracorporeal shockwave therapy is one of the modalities used to treat carpal tunnel syndrome; however, prior research has demonstrated mixed positive and negative effects of this treatment; hence its efficacy is debatable. The aim of this study was therefore to evaluate the efficacy of focused extracorporeal shockwave therapy, in terms of symptoms, hand function, and nerve parameters, in patients with moderate-to-severe carpal tunnel syndrome. Low-to-moderate-intensity focused extracorporeal shockwave therapy was administered once per week for 3 sessions. The results showed that focused extracorporeal shockwave therapy plus conservative treatment revealed short-term improvement in symptoms, hand function, and nerve conduction more effectively than conservative treatment alone.

Carpal tunnel syndrome (CTS) is the most common upper extremity entrapment neuropathy, affecting 232 per 100,000 person-years globally, with women being affected more frequently than men (1). Individuals frequently performing wrist and hand activity or those with underlying conditions, such as diabetes, hypothyroidism, pregnancy, and obesity, are more likely to develop CTS than others (2). The major symptoms of CTS are numbness, tingling sensations, or pain in the area of the median nerve distribution. Some patients may experience night-time symptoms that disturb sleep. If CTS is left untreated, the median-innervated intrinsic hand muscles may gradually atrophy and weaken (3). This impacts activities of daily living and health-related quality of life (HRQoL) (4).

The main treatments for CTS are conservative and surgical. Most patients begin with conservative treatment; however, if symptoms do not improve after full conservative treatment, surgical therapy should be considered (5). Several conservative approaches are commonly used to treat CTS, such as ultrasound, laser therapy, and wrist splinting (6–8). Nevertheless, some conventional treatments have shown inconsistent results in earlier studies. Large, long-term trials are thus required to confirm the potential benefits of these conservative treatments for CTS.

Shockwave therapy is a physical modality used for various musculoskeletal disorders (9, 10). The 2 types of shockwaves, focused extracorporeal shockwave therapy (fESWT) and radial shockwave therapy, differ in terms of their source of generators, characteristics, and properties. fESWT is primarily generated in water via electrohydraulic, electromagnetic, or piezoelectric sources. These generators produce a pressure field that converges intensely into a deep, focal area, where maximum pressure is obtained. In contrast to radial shockwaves generated by the acceleration of a projectile via a tube of compressed air, a pressure field diverges to a more superficial tissue region. Based on these factors, some research indicates that radial pressure waves are not true shockwaves (11–13).

Shockwaves promote biological changes at the molecular, cellular, and tissue levels via mechanotransduction principles, contributing to tissue healing. Previous evidence showed that fESWT can aid in lowering inflammation and alleviating pain by reducing proinflammatory cytokines. Moreover, fESWT stimulates the release of nitric oxide, resulting in vasodilatation and neovascular formation (14, 15), particularly when using low-to-moderate treatment intensities. In contrast, high treatment intensities should be used cautiously because of the risk of tissue damage (16).

Prior studies of fESWT in CTS have demonstrated improvements in symptoms, functional scores, and pain (17–20). Furthermore, low-to-moderate intensity fESWT was shown to have a favourable effect on nerve conduction studies (21, 22). Despite the beneficial effects of fESWT; however, a recent systematic review and meta-analysis indicated that the effects of shockwave therapy on individuals with CTS were not significantly different from the effects of the night wrist splint (23). Therefore, the effects of fESWT on CTS remain controversial.

To the best of our knowledge, a standard protocol for fESWT in CTS has not been currently established. Most of the aforementioned studies were conducted using relatively low-intensity fESWT, which may not be effective for treating CTS. Furthermore, no such studies of individuals with moderate-to-severe CTS have been performed. Therefore, the current study was conducted to evaluate the efficacy of low-to-medium intensity fESWT on symptoms, function, and nerve conduction in patients with moderate-to-severe CTS.

## METHODS

### Study design

The current study was a prospective, single-blind, parallel-group randomized controlled trial (RCT). Participants were recruited through the outpatient clinic of Department of Rehabilitation Medicine, Faculty of Medicine Ramathibodi Hospital, Mahidol University, Bangkok, Thailand from 1 June to 30 September 2018. The trial followed the Declaration of Helsinki and was approved by the Committee on Human Rights Related to Research involving Human Subjects of Faculty of Medicine Ramathibodi Hospital, Mahidol University, Bangkok, Thailand (MURA2017/708). All participants provided their written informed consent. Participants could stop participating in the study if they experienced any inconvenience or discomfort.

### Study population

The inclusion criteria were as follows: (*i*) participants were aged up to 75 years and had a symptom duration of up to 36 months. (*ii*) Participants had been diagnosed with CTS based on numbness in the median nerve distribution area or pain and swelling in the hand, which were frequently aggravated during the night or by repetitive use of the affected hand and were relieved by shaking the hand. A sense of hand weakness may also be present. In addition, physical examination revealed at least 1 of the following abnormalities: decreased sensation in the area of the hand supplied by the median nerve, muscle atrophy or weakness of the median nerve-innervated muscles, or a positive on either Phalen’s test or the carpal compression test (24). (*iii*) Participants had moderate-to-severe CTS based on the American Association of Neuromuscular and Electrodiagnostic Medicine (AANEM) criteria (25), diagnosed by a qualified Thai Board of Rehabilitation Medicine doctor. (*iv*) Participants had not been treated with pain medications, such as non-steroidal anti-inflammatory (NSAIDs), steroids, or anti-neuropathic drugs, within the 2 weeks before participating in the study. (*v*) Participants had provided informed consent.

The exclusion criteria were: (*i*) a history of carpal tunnel release, steroid injections, or severe infection or inflammation of the affected wrist; (*ii*) contraindications for fESWT, such as pacemaker implantation, bleeding disorders, pregnancy, or malignancy; and (*iii*) refusal to participate in the study.

### Sample size

The sample size was calculated based on the Boston Carpal Tunnel Questionnaire (BCTQ) scores, as described in a study by Vahdatpour et al. (22), with a significance level of 0.05, a power of 80%, and a difference effect size of 0.5. Assuming a 10% dropout rate, 24 participants (12 per group) were recruited for the study. The 24 participants were randomly allocated to 2 groups: an intervention group (fESWT plus conservative treatment) and a control group (conservative treatment alone). The randomization process was performed using computers, and group assignments were concealed in sealed envelopes. The participants knew which treatment group they were assigned to, but the outcome assessor was blinded to the treatment group allocation.

### Interventions

*Focused extracorporeal shockwave therapy.* The participants in the intervention group received fESWT using a DUOLITH SD1 shockwave device (Storz Medical, Tägerwilen, Switzerland). fESWT was applied perpendicular to the distal wrist crease using low-to-medium intensity, 1500 shocks per session, and 1 session per week for a total of 3 consecutive sessions. The energy flux density (EFD) was titrated from 0.01 to 0.15 mJ/mm^2^ to the maximal tolerable point of pain.

*Conservative treatment.* Both the intervention and control groups received conservative treatment, which was explained both verbally and in a brochure. This included education regarding the signs and symptoms of CTS, risk factors for CTS, behaviour modification advice, and proper activities. Participants also received a night wrist splint and performed gliding exercises for CTS (6, 26). All participants were given a logbook to record the frequency of night splint use and recommended exercises.

### Measures

*Primary outcome.* The Thai version of the Boston Carpal Tunnel Questionnaire (T-BCTQ). The BCTQ is 1 of the most widely used tools to assess the progress of symptoms and function in patients with CTS. It consists of 2 components: the symptom severity scale (BCTQs) and the functional status scale (BCTQf) (27). The original version of the BCTQ consists of 11 questions for the BCTQs (including pain, weakness, and numbness subscales), whereas the BCTQf consists of 8 questions. Each question has a score ranging from 1 to 5 points. A score of 1 indicates less severe symptom or the ability to more easily perform tasks, while a score of 5 indicates more severe symptoms or the ability to perform tasks only with difficulty. The total scores of the BCTQs and BCTQf are 55 points and 40 points, respectively. To comply with Thai culture and language, the Thai version of the BCTQ was used to evaluate hand symptoms and function in this study. Good reliability of the T-BCTQ has been reported (28).

*Secondary outcomes.* (*i*) Electrodiagnostic studies. Electrodiagnosis was performed using a Sierra^®^ Summit™ device (Cadwell industries, Kennewick, Washington, USA). The mean skin temperature in the electrodiagnostic room was maintained between 32°C and 34°C. The electrodiagnostic parameters of the median nerve, including the distal sensory latency (DSL), distal motor latency (DML), sensory nerve action potential (SNAP) amplitude, compound motor action potential (CMAP) amplitude, and area under the curve (AUC), were evaluated using a standard protocol based on AANEM. The following electrodiagnostic criteria for determining the degree of severity were as follows: (*i*) mild CTS was characterized by prolonged DSL and normal motor studies without evidence of axonal loss, (*ii*) moderate CTS was characterized by delayed DSL and DML without axonal loss, (*iii*) severe CTS was characterized by any of the above nerve conduction study abnormalities with evidence of axonal loss, as defined by either low or absent SNAP or CMAP amplitude, or the presence of an active denervation sign or chronic neurogenic pattern changes (large amplitude, long duration, or excessive polyphasics) of motor unit potentials on needle electromyography (25). (*ii*) Ultrasonography. The ultrasonography of the median nerve was performed using a Sierra^®^ Summit™ device. The median nerve cross-sectional area (CSA) was measured at the 2 levels: at the distal wrist crease (CSA_DWC_) (at the pisiform level) and the forearm level (12 cm proximal to the distal wrist crease). To determine the wrist-to-forearm area ratio (swelling ratio), the median nerve CSA at the wrist was divided by the CSA at the forearm level (29).

In the current study, all outcome measures were assessed at baseline and at 3 and 6 weeks from baseline.

### Statistical analysis

Statistical analysis was performed using R programming (https://www.r-project.org/). The data normality of the secondary outcomes, including the electrodiagnostic parameters, and the median nerve CSA were determined using the Shapiro–Wilk test. All of these outcomes were determined to have a non-normal distribution. According to the T-BCTQ, which is the categorical data and the distribution of the secondary outcomes, the Friedman test was used to analyse the changes within the same group from baseline to 3 and 6 weeks. Furthermore, the Mann–Whitney *U* test was used to compare differences between the groups at each follow-up visit. In addition, the difference between the 2 groups of the primary outcome were analysed using mixed-model analysis (shown in Table SI). *p*-values less than 0.05 were considered statistically significant.

## RESULTS

**Fig. 1:**
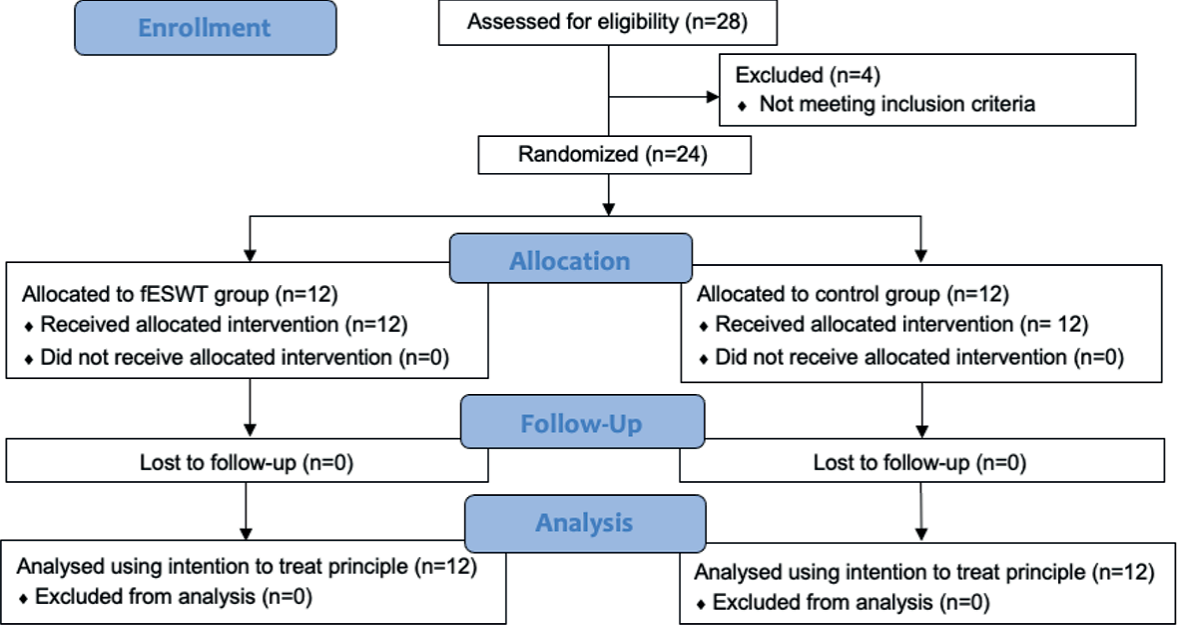
Consort diagram of the study protocol. fESWT: focused extracorporeal shockwave therapy.

A total of 24 patients diagnosed with moderate-to-severe CTS participated in the study (12 patients per group). The flow of the study protocol and the baseline demographic characteristics of the participants in the 2 groups are shown in Fig. 1 and Table I, respectively.

**Table I:** Baseline demographic and clinical characteristics of the participants

	fESWT (*n* = 12)	Control (*n* = 12)
Age, mean ± SD, years	60.25 ± 6.37	58 ± 10.49
≤60 years, *n* (%)	7 (58.33)	6 (50)
>60 years, *n* (%)	5 (41.67)	6 (50)
BMI, mean ± SD, kg/m^2^	25.47 ± 4.50	26.93 ± 4.75
Sex, *n* (%)		
Female	12 (100)	12 (100)
Dominant side, *n* (%)		
Right	12 (100)	9 (75)
Left	0	3 (25)
Lesion site, *n* (%)		
Right	4 (33.33)	4 (33.33)
Left	8 (66.67)	8 (66.67)
Severity, *n* (%)		
Moderate	6 (50)	7 (58.33)
Severe	6 (50)	5 (41.67)
Comorbid disease, *n* (%)		
Diabetes mellitus	1 (8.33)	4 (33.33)
Hypertension	4 (33.33)	7 (58.33)
Dyslipidaemia	2 (16.67)	4 (33.33)
Symptoms duration, *n* (%)		
≤1 year	8 (66.67)	10 (83.33)
>1 year	4 (33.33)	2 (16.67)
T-BCTQs score, median (IQR)	19.00 (17.00, 31.00)	19.50 (16.25, 24.25)
T-BCTQf score, median (IQR)	16.00 (14.00, 22.00)	16.50 (15.00, 19.25)
DSL, median (IQR), ms	5.30 (4.43, 5.93)	4.40 (4.20, 5.15)
SNAP amplitude, mcV, median (IQR)	14.80 (10.80, 20.40)	12.60 (6.80, 31.70)
DML, ms, median (IQR)	5.70 (5.20, 7.30)	5.60 (4.80, 6.90)
CMAP amplitude, mV, median (IQR)	5.50 (3.80, 11.70)	7.00 (5.90, 8.50)
AUC, mVms, median (IQR)	20.19 (14.58, 27.95)	19.57 (16.16, 26.27)
CSA_DWC_, mm^2^, median (IQR)	12.90 (11.90, 16.10)	13.60 (10.80, 15.30)
Swelling ratio, median (IQR)	1.66 (1.50, 2.69)	1.66 (1.48, 2.70)

fESWT: focused extracorporeal shockwave therapy; BMI: body mass index; T-BCTQs: Thai version of the Boston Carpal Tunnel Questionnaire of symptom severity; T-BCTQf: Thai version of the Boston Carpal Tunnel Questionnaire of functional status; DSL: distal sensory latency; SNAP: sensory nerve action potential; DML: distal motor latency; CMAP: compound motor action potential; AUC: area under curve; CSA: cross-sectional Area; DWC: distal wrist crease.

**Fig. 2:**
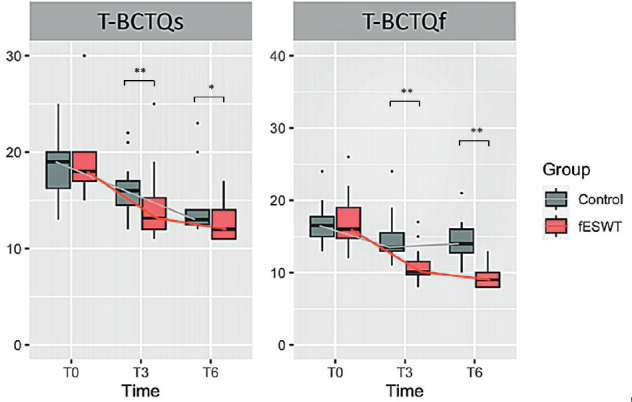
Box-plot showing the comparison of changes in Thai version of the Boston Carpal Tunnel Questionnaire of symptom severity (T-BCTQs) and Thai version of the Boston Carpal Tunnel Questionnaire of functional status (T-BCTQf ) scores between the 2 groups. (A) Changes in T-BCTQs score in the fESWT and control groups at baseline and at 3 (T3) and 6 weeks (T6) after baseline. (B) Changes in T-BCTQf score in the fESWT and control groups at baseline and at 3 and 6 weeks after baseline. Data are shown as *p < 0.05, and **p < 0.01. fESWT: focused extracorporeal shockwave therapy; T-BCTQ: Thai version of the Boston Carpal Tunnel Questionnaire.

For the overall analysis, regarding the primary outcome, almost all the T-BCTQs and T-BCTQf scores in both groups were significantly lower at 3 and 6 weeks than at baseline; with the exception of the T-BCTQs score in the control group, which reached significance only at 6 weeks from baseline. The reduction was greater in the intervention group than in the control group at all time-points. There was also a significant between-group difference in the T-BCTQs and T-BCTQf scores at 3 and 6 weeks from baseline, favouring fESWT (*p* < 0.05) (Table II and Fig. 2, respectively). The *p*-value compared between groups at each measurement time using the Mann–Whitney *U* test is shown in Table II. In addition, the mean difference between the 2 groups (95% confidence interval; 95% CI) is presented in Table SI.

**Table II:** Changes in Thai version of the Boston Carpal Tunnel Questionnaire of symptom severity (T-BCTQs), Thai version of Boston Carpal Tunnel Questionnaire of functional status (T-BCTQf), distal sensory latency (DSL), distal motor latency (DML), Sensory Nerve Action Potential (SNAP) amplitude, compound motor action potential (CMAP) amplitude, area under curve (AUC), median nerve cross-sectional area (CSA), and swelling ratio between groups

Outcome measures	fESWT group (*n* = 12) Median (IQR)	Control group (*n* = 12) Median (IQR)	*p-*value
Thai version of the Boston Carpal Tunnel Questionnaire score			
T-BCTQs			
Pre-treatment	19.00 (17.00, 31.00)	19.50 (16.25, 24.25)	
After 3 weeks	13.00 (12.00, 16.00)**	16.00 (14.25, 20.25)	0.003^b^
After 6 weeks	12.00 (11.00, 14.00)***	13.00 (12.25, 18.50)*	0.032^b^
*p-*value	< 0.001^a^	0.001^a^	
T-BCTQf			
Pre-treatment	16.00 (14.00, 22.00)	16.50 (15.00, 19.25)	
After 3 weeks	10.00 (9.00, 13.00)**	13.50 (13.00, 16.50)*	0.001^b^
After 6 weeks	9.00 (8.00, 10.00)***	14.00 (12.25, 16.00)*	0.002^b^
*p-*value	< 0.001^a^	< 0.001^a^	
Electrodiagnosis			
DSL (ms)			
Pre-treatment	5.30 (4.43, 5.93)	4.40 (4.20, 5.15)	
After 3 weeks	4.40 (4.20, 5.20)**	4.70 (4.35, 5.30)	0.005^b^
After-6 weeks	4.75 (4.13, 5.53)	4.20 (3.90, 5.25)	0.862^c^
*p-*value	0.004^a^	0.08^c^	
SNAP amplitude (mcV)			
Pre-treatment	14.80 (10.80, 20.40)	12.60 (6.80, 31.70)	
After 3 weeks	20.40 (10.30, 26.80)	20.50 (11.10, 31.50)	0.488^c^
After 6 weeks	28.50 (11.70, 32.50)**	14.70 (8.10, 26.00)	0.184^c^
*p-*value	0.001^a^	0.47^c^	
DML (ms)			
Pre-treatment	5.70 (5.20, 7.30)	5.60 (4.80, 6.90)	
After 3 weeks	5.20 (4.50, 5.90)**	5.60 (4.70, 6.30)	0.043^b^
After 6 weeks	5.30 (5.00, 5.80)	5.50 (4.50, 6.40)	0.224^c^
*p-*value	0.014^a^	0.505^c^	
CMAP amplitude (mV)			
Pre-treatment	5.50 (3.80, 11.70)	7.00 (5.90, 8.50)	
After 3 weeks	5.80 (4.60, 10.50)	8.00 (5.70, 8.80)	0.954^c^
After 6 weeks	5.50 (4.30, 9.70)	7.70 (6.10, 8.20)	0.453^c^
*p-*value	0.138^c^	0.751^c^	
AUC (mVms)			
Pre-treatment	20.19 (14.58, 27.95)	19.57 (16.16, 26.27)	
After 3 weeks	20.95 (15.28, 28.40)	23.31 (13.00, 26.61)	0.525^c^
After 6 weeks	18.81 (15.71, 26.72)	22.79 (18.58, 25.09)	0.564^c^
*p-*value	0.201^c^	0.459^c^	
Ultrasonographic CSA_median nerve			
CSA_DWC_ (mm^2^)			
Pre-treatment	12.90 (11.90, 16.10)	13.60 (10.80, 15.30)	
After 3 weeks	14.00 (11.00, 15.50)	13.70 (11.90, 17.20)	0.204^c^
After 6 weeks	11.90 (10.10, 16.40)	11.50 (10.70, 16.40)	0.326^c^
*p-*value	0.706^c^	0.516^c^	
Swelling ratio			
Pre-treatment	1.66 (1.50, 2.69)	1.66 (1.48, 2.70)	
After 3 weeks	1.59 (1.48, 2.03)	1.77 (1.27, 1.99)	0.954^c^
After 6 weeks	1.53 (1.25, 1.82)	1.47 (1.16, 1.73)	0.386^c^
*p-*value	0.558^c^	0.510^c^	

a Significant difference within the same group (Friedman test).

b Significant post-intervention difference between groups (Mann–Whitney *U* test).

c No statistically significant difference.

Data are shown as

* *p* < 0.05,

** *p* < 0.01, and

*** *p* < 0.001. fESWT: focused extracorporeal shockwave therapy; DWC: distal wrist crease.

**Fig. 3:**
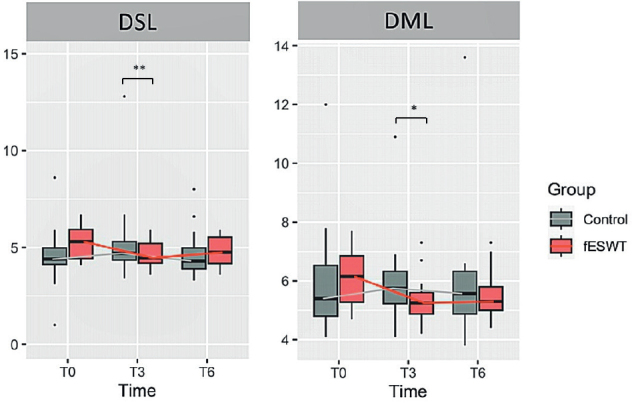
Box-plot showing the comparison of changes in distal sensory latency (DSL) and distal motor latency (DML) between the 2 groups. (A) Changes in DSL in the fESWT and control groups at baseline and at 3 (T3) and 6 weeks (T6) after baseline. (B) Changes in the DML in the fESWT and control groups at baseline and at 3 and 6 weeks after baseline. Data are shown as ns *p < 0.05, and **p < 0.01. fESWT: focused extracorporeal shockwave therapy.

Regarding secondary outcomes, compared between the 2 groups, there was also a significant difference in DSL and DML at 3 weeks from baseline, favouring fESWT (*p* < 0.05) (Fig. 3). Otherwise, no statistically significant difference was found in any of the other electrodiagnostic parameters.

For ultrasonographic measurement of the median nerve cross-sectional area, both groups demonstrated a slight increase in the median nerve CSA_DWC_ at 3 weeks and a decrease at 6 weeks compared with baseline. However, these changes were not significant at all times. Also, the swelling ratio did not change significantly at 3 and 6 weeks from baseline, either within the same group or between the 2 groups.

Furthermore, according to the demographic data (Table I), 46% of the participants in this study were over 60 years of age, and 25% of the participants had symptom durations of more than 1 year. Therefore, subgroup analyses for age and symptom duration were performed. In the subgroup analyses of (*i*) age 60 years or less and (*ii*) symptom durations equal to or less than 1 year, there was a statistically significant difference (*p* < 0.01) between groups in terms of T-BCTQs and T-BCTQf, favouring fESWT at 3 and 6 weeks from baseline. Regarding nerve recovery, DSL revealed a significant difference (*p* < 0.01) between groups at 3 weeks. Moreover, SNAP amplitude demonstrated a significant difference (*p* < 0.05) between groups at 6 weeks in the subgroups of (*i*) and (*ii*) (Tables SII and SIII, respectively). Among the final subgroup (*iii*) age over 60 years, T-BCTQf showed a statistically significant difference (*p* < 0.001) between groups at 3 and 6 weeks from baseline. These findings were consistent with the trends observed in the subgroups of (*i*) and (*ii*).Nevertheless, T-BCTQs, DSL, and SNAP amplitude showed no statistical significance between groups at all time-points (Table SIV).

No adverse events were reported during the study period in either the intervention or the control groups.

## DISCUSSION

Overall, this study showed statistically significant improvements in treatment outcomes, including the T-BCTQs, T-BCTQf, DSL, and DML, following fESWT. Nevertheless, the other electrodiagnostic parameters and the ultrasound-obtained CSA of the median nerve did not show any significant difference between the 2 groups.

### Thai version of the Boston Carpal Tunnel Questionnaire

The current study demonstrated significant between-group differences in the T-BCTSs and T-BCTQf scores at 3 and 6 weeks, favouring fESWT. This is consistent with a previous study by Vahdatpour et al. (22), which administered fESWT once per week with consecutive stepwise energy flux densities of 0.05, 0.07, 0.1, and 0.15 mJ/mm^2^ per session. While the control group received a sham fESWT plus conservative treatment, including a wrist splint, celecoxib, and vitamin B1. Their findings revealed a significant reduction in the BCTQs and BCTQf scores in the fESWT group at 3 and 6 months after treatment. Furthermore, the study also showed a statistically significant difference in the BCTQs and BCTQf scores between the groups, favouring fESWT.

In addition to this, there was previous research that used fESWT in CTS. Seok & Kim (17), Notarnicola et al. (18), Paoloni et al. (19), and Gesslbaver et al. (20) revealed statistically significant changes in the BCTQ, Levine’s severity questionnaire (LSQ), or visual analogue scale (VAS) within the same group after treatment with fESWT. However, no statistically significant differences were found between the fESWT and control groups. The reason for the lack of significance between groups may be attributable to the treatment protocol, particularly in terms of EFD and total number of sessions. Notarnicola et al. (18), Paoni et al. (19), and Gesslbaver et al. (20) used very low-intensity fESWT (EFD ranging from 0.03 to 0.05 mJ/mm^2^). By contrast, Seok & Kim (17) applied titrated EFD ranging from 0.09 to 0.29 mJ/mm^2^ to the maximally tolerable pain level for only a single treatment session. Either a very low intensity or a small total number of sessions may have been inadequate to demonstrate the positive treatment effects of fESWT. In addition to these factors, the total amount of energy delivered from all treatment sessions, or the accumulated dose, may also affect the efficacy of fESWT, with a lower accumulated dosage potentially resulting in a less favourable outcome.

### Electrodiagnostic parameters

This study revealed a significant between-group difference in the DSL and DML at 3 weeks from baseline, with the fESWT group exhibiting a greater reduction. This is consistent with the findings of Gesslbaver et al. and Vahdatpour et al., who demonstrated an improvement in nerve conduction following fESWT. Gesslbaver et al. in 2021 (20) applied low-intensity fESWT (0.05 mJ/mm^2^) once weekly for 3 weeks and observed a significant between-group difference in the DSL at 3 weeks following fESWT. Another study by Vahdatpour et al. in 2016 (22) used the low-to-moderate intensity fESWT, and found a statistically significant difference in the DSL and DML between the groups at 3 and 6 months after fESWT. However, these 2 studies did not investigate the treatment effect on nerve amplitude. According to the results from both previous studies and the current study, low-to-moderate intensity fESWT may have a beneficial effect on nerve latency or the myelinated component.

In terms of axonal regeneration, as measured by nerve amplitude, the current study revealed an increase in SNAP amplitude in the fESWT group, while CMAP amplitude remained unchanged within the same group. However, no statistically significant difference was found between the 2 groups. Compared with previous studies, Soek et al. (17) applied a single session of fESWT (EFD ranged from 0.09 to 0.29 mJ/mm^2^). After treatment, there was no significant difference in nerve amplitude between the fESWT and the control group that received local corticosteroid injection (LCI). Furthermore, another two studies (30, 31) applied radial shockwave therapy once a week for three weeks consecutively. The control group received LCI and a night wrist splint. They found no significant differences between the groups in terms of nerve amplitude.

Based on these previous results and the results from the current study together, it becomes uncertain whether ESWT may influence axonal regeneration in humans. Further research in humans is required to ascertain the optimal protocol for ESWT in the context of axonal regeneration.

### Ultrasonographic measurement of cross-sectional area of the median nerve

It was anticipated that the anti-inflammatory effect of fESWT would reduce venous congestion and edema of the median nerve, thereby reducing the median nerve CSA. However, the current study did not reveal a significant decline in the median nerve CSA between the groups. Notably, the current study measured only the nerve circumference, which may not reflect all sonographic nerve features. Other sonographic features, such as fascicular texture and nerve echogenicity, should be evaluated to more fully investigate nerve recovery. In addition, nerve vascularity should be examined by colour Doppler ultrasound (32). Ke et al. (33) investigated the effect of 3 sessions of radial shockwave therapy on the median nerve CSA in patients with mild-to-moderate CTS. They showed a significant improvement in the median nerve CSA at 14 weeks from baseline compared with that of the sham shockwave group. However, whether the different characteristics of focused or radial shockwaves can affect ultrasonographic feature changes remains unclear. Therefore, future studies should compare the effects of fESWT vs radial shockwave therapy on the median nerve CSA in patients with CTS.

There are several postulated explanations for the beneficial effects of fESWT on symptomatic relief, functional improvement, and nerve recovery. In terms of symptomatic relief, fESWT provides anti-inflammatory and analgesic effects via various mechanisms, such as the gate control theory, suppression of proinflammatory cytokines, and destruction of unmyelinated C-fibres. This is supported by an *in vitro* study in which fESWT enhanced neuronal nitric oxide synthase activity, thereby increasing nitric oxide levels (15). Once nitric oxide reaches the optimal level, it will induce vasodilatation, angiogenesis, and neurotransmission improvement. Moreover, they also showed that mixed inflammatory cytokines, such as tumour necrosis factor-alpha and interferon-gamma, were suppressed 30–60 min after fESWT application. Another study investigated the effect of fESWT on the distal femurs of rat models. The results showed that fESWT significantly diminished immunoreactive neurones for substance P and calcitonin gene-related peptide within the dorsal root ganglion and reduced unmyelinated C-fibres in the femoral nerve, hence interfering with pain signal transmission (34–36). The analgesic effect of fESWT is also supported by a study performed by García-Muntión et al. (37). The authors showed that fESWT significantly increased the pressure pain threshold (PPT), especially for the moderate pain-generating intensity of fESWT.

Regarding nerve recovery, fESWT contributes to an improvement of nerve latency that may reflect the remyelination process, as mentioned above. This effect is supported by a study by Hausner et al. (21), who applied fESWT to rat models with injured sciatic nerves. The histological finding revealed a significantly increased number of myelinated fibres at 3 weeks after treatment. In addition, there was a significant improvement in gait analysis as shown by an increase in the stance time and a decrease in the swing time, which may indicate a more stable gait pattern of the rat models. Another study by Yahata et al. (38) explored the effects of fESWT on rat models with spinal cord injury. The authors found that focused shockwave resulted in significant improvements in locomotor function, mechanical allodynia, and thermal allodynia. The shockwave group also demonstrated a considerable increase in vascular endothelial growth factor, which is crucial for new blood vessel formation and nerve repair.

### Limitations

The current study has some limitations. First, the majority of the participants in this study had moderate-to-severe CTS. Therefore, the severity of their conditions may have influenced the improvement in functional outcomes as well as nerve regeneration after treatment with ESWT. Secondly, diabetes mellitus (DM) was presented in 5 of the cases (1 in fESWT and 4 in controls) in this study. Diabetes may contribute to adverse effects on symptoms and treatment responses in patients with CTS. However, the HbA1c levels of those 5 patients were less than 7%, indicating that their diabetes was well controlled. Thirdly, as previously mentioned regarding the subgroup analyses for age and symptom durations (in the results section), those 2 factors may have an impact on treatment outcomes, especially symptom and nerve recovery. Therefore, further studies with a larger sample size that stratifies subjects based on age and symptom durations should be conducted to determine the influence of these factors. Fourthly, the current study had no long-term follow-up. Future research should be undertaken over a longer period of time to evaluate the long-term effects of fESWT.

### Conlusion

fESWT demonstrated significant benefits with respect to short-term symptomatic relief, improved function, and improved nerve conduction in patients with moderate-to-severe CTS. Moreover, the results of this study add to the current evidence regarding the clinical application of fESWT. These results will increase confidence in a conservative treatment option, especially for patients with moderate-to-severe CTS who have declined surger, are awaiting surgery, or prefer conservative treatment.

## Supplementary Material

EFFICACY OF FOCUSED SHOCKWAVE THERAPY IN PATIENTS WITH MODERATE-TO-SEVERE CARPAL TUNNEL SYNDROME: A PRELIMINARY STUDY
