# Supplementary material for: EFFICACY OF FOCUSED SHOCKWAVE THERAPY IN PATIENTS WITH MODERATE-TO-SEVERE CARPAL TUNNEL SYNDROME: A PRELIMINARY STUDY
Source: J Rehabil Med. 2024 Feb 8;56:13411. doi: 10.2340/jrm.v56.13411 (PMC10865893; doi:10.2340/jrm.v56.13411)
Supplement: EFFICACY OF FOCUSED SHOCKWAVE THERAPY IN PATIENTS WITH MODERATE-TO-SEVERE CARPAL TUNNEL SYNDROME: A PRELIMINARY STUDY [file JRM-56-13411-s1.pdf]

Supplementary material has been published as submitted. It has not been copyedited, typeset or checked for scientific content by Journal of Rehabilitation Medicine

Table SI. Overall analysis (n = 24).
Compared changes from baseline to each follow-up visit between the fESWT and control groups (analyzed by mixed model analysis)

Outcome measures	Mean difference (95%CI)	*p*-value
**T-BCTQ score**		
T-BCTQs		
Baseline to 3 weeks	-5.52 (-9.29 – -1.76)	0.004
Baseline to 6 weeks	-5.67 (-9.37 – -1.96)	0.003
T-BCTQf		
Baseline to 3 weeks	-4.18 (-5.75 – -2.61)	< 0.001
Baseline to 6 weeks	-5.08 (-6.63 – -3.54)	< 0.001
**Electrodiagnosis**		
DSL (ms)		
Baseline to 3 weeks	-1.07 (-1.82 – -0.33)	0.005
Baseline to 6 weeks	-0.79 (-1.54 – -0.06)	0.035
SNAP amplitude (mcV)		
Baseline to 3 weeks	1.14 (-7.56 – 9.84)	0.789
Baseline to 6 weeks	8.15 (-0.42 – 16.72)	0.269
DML (ms)		
Baseline to 3 weeks	-0.73 (-1.39 – -0.07)	0.031
Baseline to 6 weeks	-0.54 (-1.21 – 0.12)	0.106
CMAP amplitude (mV)		
Baseline to 3 weeks	0.13 (-0.86 – 1.12)	0.717
Baseline to 6 weeks	-0.28 (-1.27 – 0.71)	0.453
AUC (mVms)		
Baseline to 3 weeks	0.23 (-4.55 – 5.02)	0.428
Baseline to 6 weeks	-2.03 (-6.67 – 2.59)	0.460
**Ultrasound median nerve CSA**		
CSA_{DWC} (mm^2)		
Baseline to 3 weeks	-1.04 (-3.23 – 1.13)	0.185
Baseline to 6 weeks	0.54 (-1.64 – 2.72)	0.918
Swelling ratio		
Baseline to 3 weeks	-0.05 (-0.45 – 0.35)	0.739
Baseline to 6 weeks	0.17 (-0.24 – 0.57)	0.479

fESWT, focused extracorporeal shockwave therapy; T-BCTQ, Thai version of the Boston Carpal Tunnel Questionnaire; T-BCTQs, Thai version of the Boston Carpal Tunnel Questionnaire of symptom severity; T-BCTQf, Thai version of Boston Carpal Tunnel Questionnaire of functional status; DSL, distal sensory latency; SNAP, Sensory Nerve Action Potential; DML, distal motor latency; CMAP, compound motor action potential; AUC, area under curve; CSA, cross-sectional area; DWC, distal wrist crease.

Table SII. Subgroup analysis for age ≤ 60 years (n = 13).

Compared changes from baseline to each follow-up visit between the fESWT and control groups (analyzed by mixed model analysis)

Outcome measures	Mean difference (95%CI)	*p*-value
**T-BCTQ score**		
T-BCTQs		
Baseline to 3 weeks	-7.57 (-13.17 – -1.97)	0.008
Baseline to 6 weeks	-7.88 (-13.48 – -2.28)	0.006
T-BCTQf		
Baseline to 3 weeks	-5.02 (-7.51 – -2.53)	< 0.001
Baseline to 6 weeks	-5.08 (-8.28 – -3.29)	< 0.001
**Electrodiagnosis**		
DSL (ms)		
Baseline to 3 weeks	-1.81 (-3.01 – -0.62)	0.003
Baseline to 6 weeks	-1.55 (-2.79 – -0.29)	0.015
SNAP amplitude (mcV)		
Baseline to 3 weeks	3.25 (-7.42 – 13.92)	0.551
Baseline to 6 weeks	10.82 (0.15 – 21.49)	0.047
DML (ms)		
Baseline to 3 weeks	-0.72 (-1.74 – -0.31)	0.168
Baseline to 6 weeks	-0.79 (-0.26 – 1.84)	0.143
CMAP amplitude (mV)		
Baseline to 3 weeks	1.13 (-0.21 – 2.47)	0.097
Baseline to 6 weeks	0.81 (-0.58 – 2.19)	0.252
AUC (mVms)		
Baseline to 3 weeks	0.97 (-6.67 – 8.62)	0.803
Baseline to 6 weeks	-2.38 (-10.02 – 5.26)	0.542

fESWT, focused extracorporeal shockwave therapy; T-BCTQ, Thai version of the Boston Carpal Tunnel Questionnaire; T-BCTQs, Thai version of the Boston Carpal Tunnel Questionnaire of symptom severity; T-BCTQf, Thai version of Boston Carpal Tunnel Questionnaire of functional status; DSL, distal sensory latency; SNAP, Sensory Nerve Action Potential; DML, distal motor latency; CMAP, compound motor action potential; AUC, area under curve; CSA, cross-sectional area; DWC, distal wrist crease.

Table SIII. Subgroup analysis for symptom duration ≤ 1 year (n = 18).

Compared changes from baseline to each follow-up visit between the fESWT and control groups (analyzed by mixed model analysis)

**Outcome measures**	**Mean difference (95%CI)**	***p*-value**
**T-BCTQ score**		
T-BCTQs		
Baseline to 3 weeks	-7.08 (-11.53 – -2.62)	0.002
Baseline to 6 weeks	-8.05 (-12.50 – -3.59)	<0.001
T-BCTQf		
Baseline to 3 weeks	-4.65 (-6.32 – -2.98)	< 0.001
Baseline to 6 weeks	-6.05 (-7.72 – -4.38)	< 0.001
**Electrodiagnosis**		
DSL (ms)		
Baseline to 3 weeks	-1.22 (-2.17 – -0.27)	0.012
Baseline to 6 weeks	-0.84 (-1.80 – 0.13)	0.090
SNAP amplitude (mcV)		
Baseline to 3 weeks	2.78 (-6.02 – 11.58)	0.536
Baseline to 6 weeks	10.29 (1.49 – 19.09)	0.022
DML (ms)		
Baseline to 3 weeks	-0.50 (-1.33 – -0.32)	0.233
Baseline to 6 weeks	-0.46 (-1.30 – 0.39)	0.288
CMAP amplitude (mV)		
Baseline to 3 weeks	0.49 (-0.67 – 1.64)	0.412
Baseline to 6 weeks	0.07 (-1.11 – 1.25)	0.910
AUC (mVms)		
Baseline to 3 weeks	-0.79 (-6.57 – 4.99)	0.788
Baseline to 6 weeks	-3.06 (-8.73 – 2.62)	0.291

fESWT, focused extracorporeal shockwave therapy; T-BCTQ, Thai version of the Boston Carpal Tunnel Questionnaire; T-BCTQs, Thai version of the Boston Carpal Tunnel Questionnaire of symptom severity; T-BCTQf, Thai version of Boston Carpal Tunnel Questionnaire of functional status; DSL, distal sensory latency; SNAP, Sensory Nerve Action Potential; DML, distal motor latency; CMAP, compound motor action potential; AUC, area under curve; CSA, cross-sectional area; DWC, distal wrist crease.

Table SIV. Subgroup analysis for age > 60 years (n = 11).

Compared changes from baseline to each follow-up visit between the fESWT and control groups (analyzed by mixed model analysis)

**Outcome measures**	**Mean difference (95%CI)**	***p*-value**
**T-BCTQ score**		
T-BCTQs		
Baseline to 3 weeks	-1.95 (-4.14 – 0.23)	0.079
Baseline to 6 weeks	-2.07 (-4.15 – 0.02)	0.052
T-BCTQf		
Baseline to 3 weeks	-3.02 (-4.53 – -1.49)	< 0.001
Baseline to 6 weeks	-4.20 (-5.65 – -2.75)	< 0.001
**Electrodiagnosis**		
DSL (ms)		
Baseline to 3 weeks	-0.44 (-0.88 – -0.00)	0.050
Baseline to 6 weeks	-0.23 (-0.66 – 0.12)	0.280
SNAP amplitude (mcV)		
Baseline to 3 weeks	-1.39 (-15.66 – 12.88)	0.849
Baseline to 6 weeks	5.66 (-7.99 – 19.32)	0.416
DML (ms)		
Baseline to 3 weeks	-0.79 (-1.51 – -0.07)	0.032
Baseline to 6 weeks	-0.39 (-1.08 – 0.29)	0.260
CMAP amplitude (mV)		
Baseline to 3 weeks	-1.12 (-2.35 – 0.11)	0.074
Baseline to 6 weeks	-1.49 (-2.67 – -0.32)	0.013
AUC (mVms)		
Baseline to 3 weeks	-1.24 (-4.72 – 2.23)	0.483
Baseline to 6 weeks	-2.46 (-5.67 – 0.74)	0.132

fESWT, focused extracorporeal shockwave therapy; T-BCTQ, Thai version of the Boston Carpal Tunnel Questionnaire; T-BCTQs, Thai version of the Boston Carpal Tunnel Questionnaire of symptom severity; T-BCTQf, Thai version of Boston Carpal Tunnel Questionnaire of functional status; DSL, distal sensory latency; SNAP, Sensory Nerve Action Potential; DML, distal motor latency; CMAP, compound motor action potential; AUC, area under curve; CSA, cross-sectional area; DWC, distal wrist crease.
